# Non-biofilm-forming *Staphylococcus epidermidis* planktonic cell supernatant induces alterations in osteoblast biological function

**DOI:** 10.1038/s41598-024-51899-7

**Published:** 2024-01-20

**Authors:** Itzia Sidney Gómez-Alonso, Gabriel Betanzos-Cabrera, Martha Cecilia Moreno-Lafont, Mario Eugenio Cancino-Diaz, Blanca Estela García-Pérez, Juan Carlos Cancino-Diaz

**Affiliations:** 1 Departamento de Microbiología, Escuela Nacional de Ciencias Biológicas, Instituto Politécnico Nacional, Manuel Carpio, Plutarco Elías Calles, Miguel Hidalgo, 11350 Mexico City, Mexico; 2 Área Académica de Nutrición, Instituto de Ciencias de la Salud, Universidad Autónoma del Estado de Hidalgo, Carretera Pachuca-Actopan Camino a Tilcuautla S/N., Pueblo San Juan Tilcuautla, 42160 Pachuca Hidalgo, Mexico; 3 Departamento de Inmunología, Escuela Nacional de Ciencias Biológicas, Instituto Politécnico Nacional, Manuel Carpio, Plutarco Elías Calles, Miguel Hidalgo, 11350 Mexico City, Mexico

**Keywords:** Bacterial infection, Biofilms

## Abstract

Staphylococcal biofilms significantly contribute to prosthetic joint infection (PJI). However, 40% of *S. epidermidis* PJI isolates do not produce biofilms, which does not explain the role of biofilms in these cases. We studied whether the supernatant from planktonic *S. epidermidis* alters osteoblast function. Non-biofilm-forming *S. epidermidis* supernatants (PJI^−^ clinical isolate, healthy skin isolate (HS), and ATCC12228 reference strain) and biofilm-forming supernatants (PJI^+^ clinical isolate, ATCC35984 reference strain, and *Staphylococcus aureus* USA300 reference strain) were included. Osteoblasts stimulated with supernatants from non-biofilm-forming isolates for 3, 7, and 14 days showed significantly reduced cellular DNA content compared with unstimulated osteoblasts, and apoptosis was induced in these osteoblasts. Similar results were obtained for biofilm-forming isolates, but with a greater reduction in DNA content and higher apoptosis. Alkaline phosphatase activity and mineralization were significantly reduced in osteoblasts treated with supernatants from non-biofilm-forming isolates compared to the control at the same time points. However, the supernatants from biofilm-forming isolates had a greater effect than those from non-biofilm-forming isolates. A significant decrease in the expression of ATF4, RUNX2, ALP, SPARC, and BGLAP, and a significant increase in RANK-L expression were observed in osteoblasts treated with both supernatants. These results demonstrate that the supernatants of the *S. epidermidis* isolate from the PJI^−^ and HS (commensal) with a non-biofilm-forming phenotype alter the function of osteoblasts (apoptosis induction, failure of cell differentiation, activation of osteoblasts, and induction of bone resorption), similar to biofilm-forming isolates (PJI^+^, ATCC35984, and *S. aureus* USA300), suggesting that biofilm status contributes to impaired osteoblast function and that the planktonic state can do so independently of biofilm production.

## Introduction

Bone is constantly remodeled, and the cells involved in this process are osteoblasts, osteoclasts, and osteocytes^1^. During bone homeostasis, bone is reabsorbed by osteoclasts and replaced with fresh bone by osteoblasts. This process involves osteoclasts promoting bone resorption indirectly by regulating osteoblast activity through the production of two cytokines, the receptor activator of NF-kB ligand (RANK-L) and osteoprotegerin (OPG). RANK-L production by osteoblasts promotes osteoclastic activity, and OPG expression blocks this activity^2–4^. Subsequently, in the resorbed zone, osteoblasts are deposited and differentiate into osteocytes, calcifying the bone matrix and promoting new bone growth^2–4^. Bacterial infections can trigger the pathological remodeling of bone, inducing the production of RANK-L in osteoblasts and decreasing the expression of OPG, which causes RANK-L to bind to its receptor RANK present on osteoclasts and induce their differentiation and maturation, contributing to bone loss, which is a typical feature of chronic orthopedic infection^5,6^.

Osteomyelitis is an inflammatory process that results in bone destruction and damage to surrounding tissues. Osteomyelitis is commonly caused by hematogenous microorganisms that reach the bone from a contagious infection or directly from trauma, surgery, or medical implants. Implant-associated osteomyelitis, such as prosthetic joint infection (PJI), is of high frequency. PJI is 1–9% common in all hip or knee arthroplasty patients^7,8^, and treatment success ranges from 30 to 90%^9^. *Staphylococcus aureus*, *Staphylococcus epidermidis*, and other coagulase-negative staphylococci (CNS) are responsible for 50% of PJI cases^10^, and in chronic PJI *S. aureus* and *S. epidermidis* are the most incidence^11^.

Bacteria that infect bones can evade the immune system through different strategies, such as biofilm formation, which makes bacteria recalcitrant towards conventional antimicrobial treatment and contributes to the incidence of infectious relapse^12,13^. Biofilms are bacterial agglomerations attached to a surface and enveloped in a polymeric extracellular matrix produced by bacteria^14,15^. Bacterial biofilm formation is associated with the development of chronic infections, such as osteomyelitis^16^, and is present within the infected bone^17,18^ and in the synovial fluid of PJI^19^. Furthermore, *S. aureus* isolates from patients with osteomyelitis and PJI can form biofilms in vitro^20–23^, as can *S. epidermidis* isolates^24^. This property confers antibiotic resistance compared to planktonic bacteria, which influences the outcome of PJI treatment management^25^.

Staphylococcal biofilms play a critical role in PJI, and the first immune cells to be recruited at the interface of a biofilm with host cells are polymorphonuclear neutrophils (PMNs)^26^. In PJI, the interaction of PMNs and granulocytic myeloid-derived suppressor cells (G-MDSCs) favors periprosthetic osteolysis. Moreover, inducing the production of proinflammatory cytokines and chemokines by PMNs promotes osteogenesis^27^. Furthermore, RANK-L induction by osteoblasts^28^ and PMNs^29,30^ promotes bone resorption by activating osteoclast functions. This inflammatory microenvironment can remove the staphylococcal biofilm, increasing the microbicidal properties of PMNs through NET formation and the release of migration inhibitory factor-related MRP-14^31^. In contrast, G-MDSCs in PJI^32^ regulate the inflammatory process through IL-10 production, which favors the persistence of staphylococcal biofilm^33^. However, the biofilm sensitivity to the microbicidal effects of PMNs varies among strains. In contrast, the biofilm of *S. aureus* is more sensitive to PMNs attack, and the biofilm of *S. epidermidis* restricts its function^34^.

The effects of staphylococcal species on osteoblasts were evaluated in vitro. The interaction of *S. aureus* with osteoblasts has demonstrated that Staphylococcal protein A is responsible for direct binding to osteoblast cells, which causes the inhibition of osteoblast proliferation and differentiation, in addition to inducing apoptosis^5,35–37^. However, the effects induced by *S. epidermidis* are poorly understood because protein A is absent in this species. Other studies have provided signals within the biofilm mechanism contributing to bone loss^13,16,17^, as is the case with supernatants obtained from *S. aureus* USA300 biofilms affecting osteoblast viability, as well as induction of apoptosis, lack of cell differentiation, and RANK-L expression^22^, which has also been observed in the *S. epidermidis* RP62A (ATCC 35984) strain with a biofilm-forming phenotype^38,39^. Currently, the effect of biofilms on osteoblasts has only been studied in laboratory-type strains^22,38,39^, and clinical isolates have not been studied. Moreover, although staphylococcal isolates of PJI are biofilm-forming in vitro, not all isolates have this phenotypic characteristic since approximately 30–40% of isolates from patients with PJI are non-biofilm-forming^40^, and the patients have the same clinical complications as those with biofilm-forming isolates. Therefore, it is unknown how these non-biofilm-forming isolates develop the disease, because the biofilm of *S. epidermidis* is the main virulence factor^12^. Therefore, this study aimed to elucidate the effect of supernatants of *S. epidermidis* isolates from patients with PJI with biofilm-forming and non-biofilm-forming phenotypes on human osteoblasts.

## Results

### Biofilm formation of the isolates

**Figure 1:**
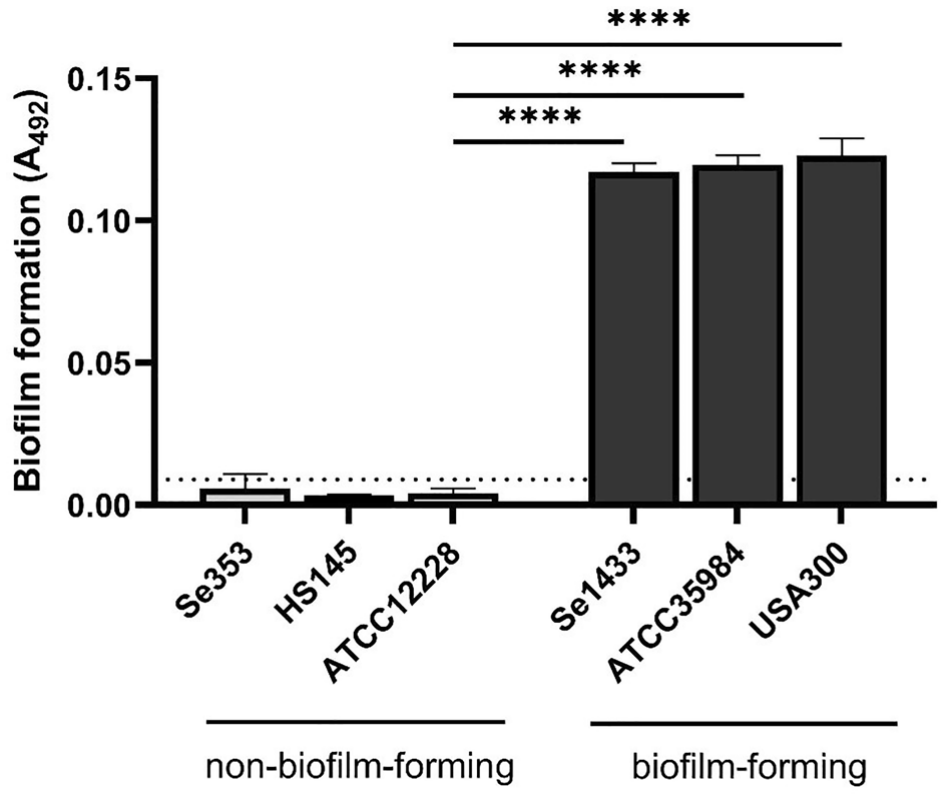
Biofilm formation among the isolates studied. Biofilm formation was determined by Christensen’s method. The dotted line indicates the cut-off value represented as 2 × the standard deviation of ATCC12228 strain (non-biofilm-forming reference strain). ****Statistically significant difference (p < 0.0001). One-way ANOVA with Dunnett’s post-test was used to compare the biofilm-forming strains with the ATCC12228 reference strain.

*S. epidermidis* isolates Se1433 and Se353 from patients with PJI, healthy skin isolate HS145, and the reference strains were studied for biofilm formation. The clinical isolate Se1433 showed biofilm formation similar to that of *S. epidermidis* strain ATCC35984 (biofilm-forming strain) and *S. aureus* USA300 (biofilm-forming clinical strain). In contrast, the clinical isolates Se353, HS145, and ATCC12228 did not show biofilm formation. Biofilm formation by the biofilm-forming strain was statistically significant compared to the ATCC12228 reference strain (non-forming biofilm strain) (Fig. 1).

### Cytotoxicity of isolate supernatants

The total protein concentration of the supernatant obtained from the TSB medium was determined. To determine the cytotoxic effect of supernatants, the concentrations of 0.01, 0.1, 1, and 2 µg/mL of total protein were tested. After 24 h of exposure, the concentrations of the supernatants did not show cytotoxicity in osteoblasts compared to the control group (p > 0.05; Supplementary Fig. 1). Based on this result, 0.1 and 1 µg/mL were used for the subsequent experiments.

### Staphylococcus planktonic cell supernatant reduces osteoblast viability by activation of apoptosis

**Figure 2:**
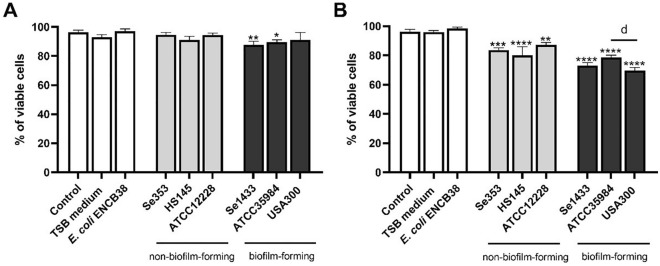
Effect of bacterial supernatants on the viability of osteoblasts. Osteoblasts (cell line MG-63) grown under non-osteogenic conditions were treated with different supernatant concentrations of (**A**) 0.1 and (**B**) 1 µg/mL for 48 h. The cells were labeled with a fluorescent marker. Osteoblasts that were untreated with the supernatant (control group) were considered 100% viable cells. The asterisk indicates that an ANOVA analysis was performed with Dunnett’s test to compare treatments with the control (*p < 0.05, **p < 0.005, ***p < 0.0005, ****p < 0.0001); a Tukey’s multiple comparison was performed to compare among treatments (letter d: p < 0.05).

In a semi-quantitative osteoblast cell viability assay (fluorescence staining), it was observed that during stimuli with 0.1 µg/mL total protein, only supernatants from isolates with the biofilm-forming phenotype (Se1433, ATCC35984) caused a reduction in cell viability compared to the control (untreated osteoblasts) (Fig. 2A). Increased reduction in viability was observed at 1 µg/mL total protein concentration of the supernatant of non-biofilm-forming isolates (Se353, HS145, and ATCC12228) relative to the control, for biofilm-forming isolates (Se1433, ATCC35984 and *S. aureus* USA300 strains), the supernatants caused a higher reduction in cell viability (Fig. 2B). Overall, this indicated a reduction in osteoblast viability by the supernatants of the non-biofilm-forming and biofilm-forming isolates.

**Figure 3:**
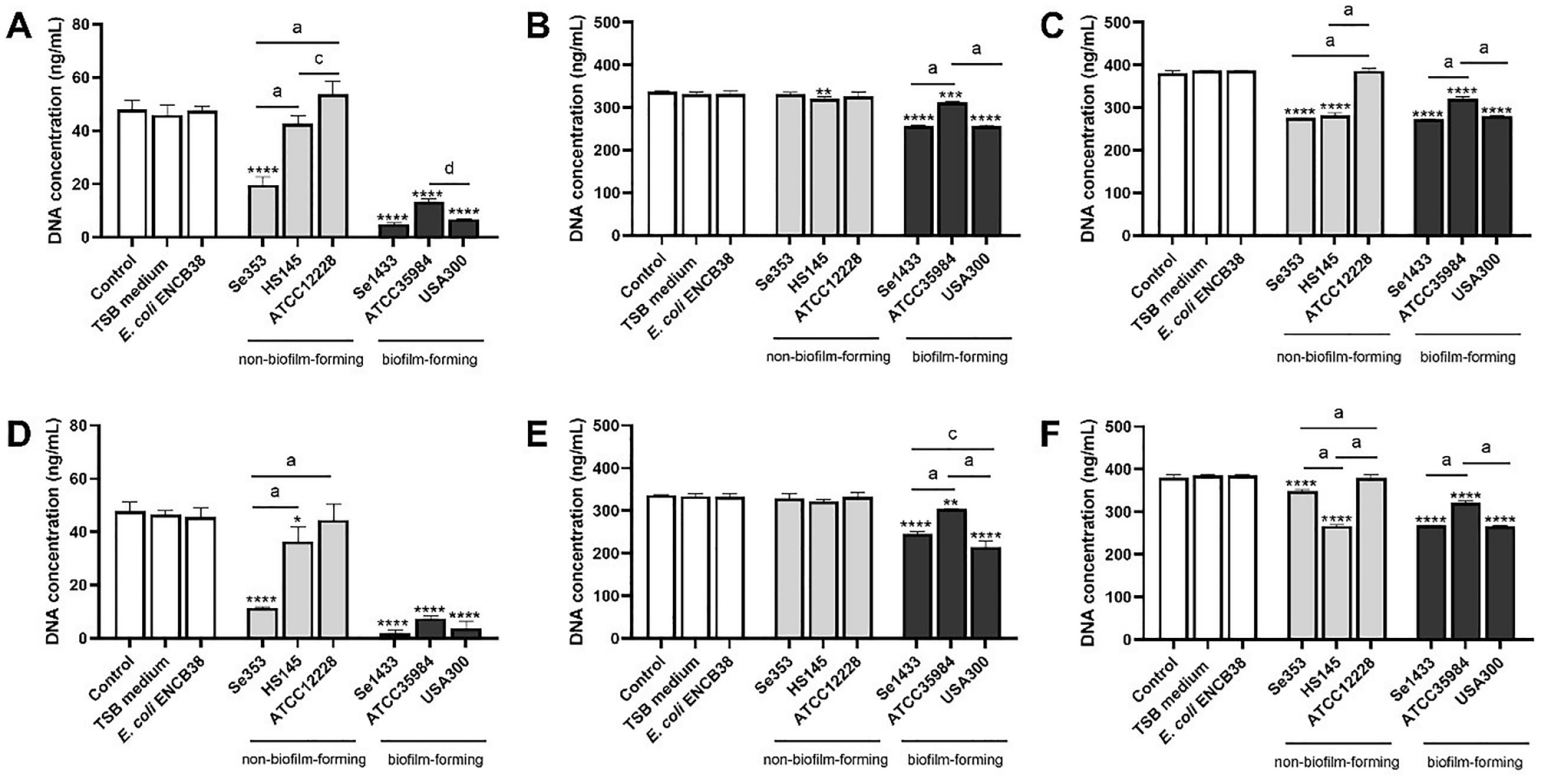
DNA quantification of osteoblasts treated with supernatant. Osteoblasts (cell line MG-63) grown under non-osteogenic conditions were treated with supernatant protein concentrations of 0.1 (**A–C**) and 1 µg/mL (**D–F**) at 3 (**A,D**), 7 (**B,E**) and 14 (**C,F**) days. Untreated osteoblasts were used as the control. After treatment, DNA content in the cells was calculated with CyQUANT kit. The asterisk indicates that an ANOVA analysis was performed with Dunnett’s test to compare treatments with the control (*p < 0.05, **p < 0.005, ***p < 0.0005, ****p < 0.0001); a Tukey’s multiple comparison was performed to compare among treatments (letters; a: p < 0.0001; c: p < 0.005).

The DNA content of osteoblasts on different days was calculated to determine the reduction in cell viability at later time points. Concerning the non-biofilm-forming isolates on day 3, only isolate Se353 had a lower DNA concentration in osteoblasts treated with supernatant at a total protein concentration of 0.1 and 1 µg/mL concerning the control (Fig. 3A,D). On day 7, there was no change in DNA content, except for HS145 isolate (Fig. 3B,E), and on day 14, only isolates Se353 and HS145 showed significantly reduced DNA content (Fig. 3C,F). In contrast, in the biofilm-forming isolates, there was a significant difference between the different supernatants at the three-time points tested, with supernatant Se1433 showing the highest reduction in DNA content. The ATCC12228 strain, TSB medium, and the supernatant of *Escherichia coli* did not show significant changes in the DNA content of the osteoblasts (Fig. 3A–F).

These results indicate that the presence of compounds released by bacterial growth in biofilm or non-biofilm conditions of staphylococci caused a significant reduction in osteoblast cell viability, and the Se1433 supernatant had the greatest viability reduction effect compared to the Se353 and HS145 supernatants.

**Figure 4:**
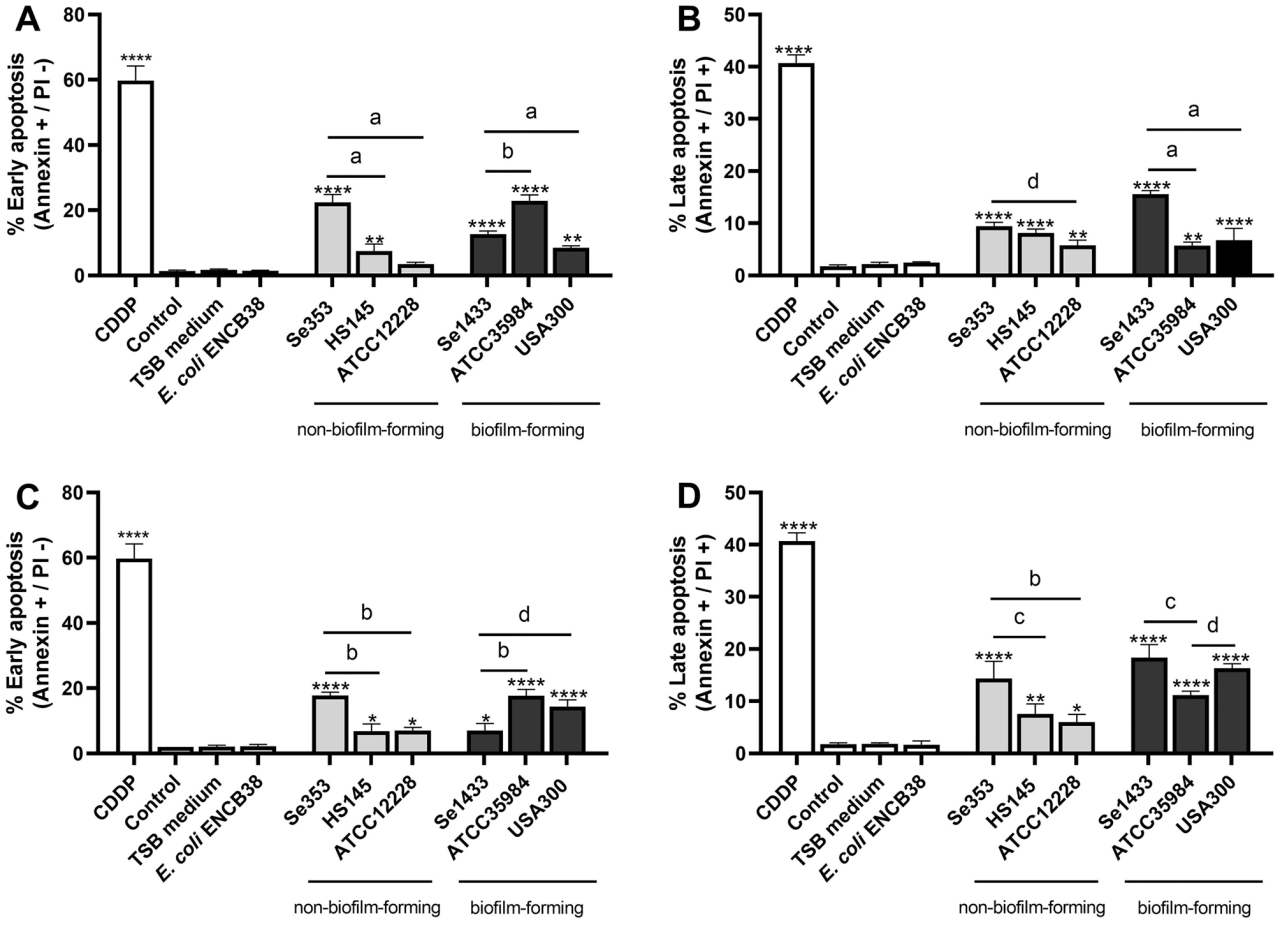
Apoptosis of osteoblasts treated with supernatant. Osteoblasts (cell line MG-63) grown under non-osteogenic conditions were treated with supernatants in concentration of 0.1 (**A,B**) and 1 µg/mL of total protein (**C,D**) after 48 h to exposure. Osteoblasts not treated with the supernatant were used as control. Osteoblasts treated with 50 µM cisplatin (CDDP) were used as positive apoptosis. Early apoptosis percentages (**A,C**) and late (**B,D**) were determined. The asterisk indicates that an ANOVA analysis was performed with Dunnett’s test to compare treatments with the control (*p < 0.05, **p < 0.005, ***p < 0.0005, ****p < 0.0001); a Tukey’s multiple comparison was performed to compare among treatments (letters; a: p < 0.0001; b: p < 0.0005; c: p < 0.005; d: p < 0.05).

The reduction in osteoblast cell viability by the different supernatants indicated that a population of cells was not involved in cell division, suggesting that apoptosis could have occurred. Apoptosis analysis of the osteoblasts treated with the supernatant was performed. After 48 h, CDPP-treated osteoblasts (positive control for apoptosis) showed an increase in early and late apoptotic states (approximately 60 and 40%, respectively). The both supernatant protein concentrations (0.1 and 1 µg/mL) of the non-biofilm-forming isolates, except for strain ATCC1228, induced early apoptosis (between 13 and 20%) compared to the control (Fig. 4A,C), similarly late apoptosis was also significantly increased compared to the control (Fig. 4B,D). The biofilm-forming isolates induced early and late apoptosis in osteoblasts treated with the supernatants at both total protein concentrations (Fig. 4A–D). However, a significant difference was observed among the different treatments, with less early apoptosis in the treatment with the Se1433 supernatant and *S. aureus* USA300, but higher late apoptosis in the Se1433 supernatant. The results showed that the supernatants from the non-biofilm-forming isolates (Se353 and HS145) drove them toward early or late apoptosis.

### Staphylococcus planktonic cell supernatant alters the osteogenic process in vitro

Osteoblasts are induced in vitro to undergo osteogenic differentiation. Alkaline phosphatase (ALP) concentration and calcium deposition (mineralization) in osteoblasts were used as phenotypic markers of osteogenic differentiation.

Osteoblast differentiation was monitored by measuring ALP levels. In untreated osteoblasts, there was an increase in the ALP content on days 3 and 7; however, on day 14, the osteoblasts maintained the same amount of ALP, suggesting that in vitro cell differentiation was achieved.

**Figure 5:**
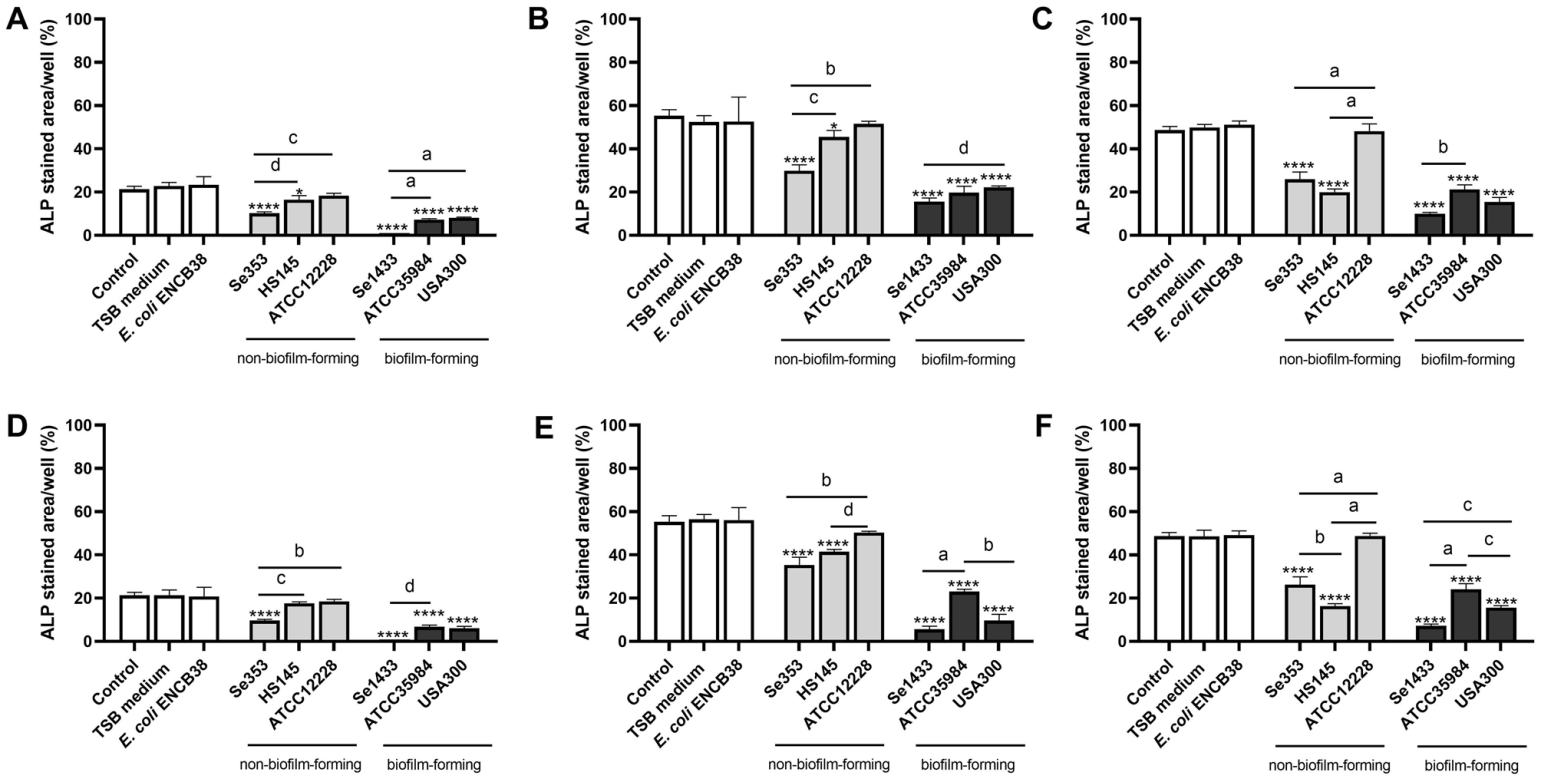
Alkaline phosphatase (ALP) activity in osteoblasts treated with Staphylococcal supernatants. Osteoblasts (cell line MG-63) were grown under osteogenic conditions and treated with supernatant protein concentrations of 0.1 (**A–C**) and 1 µg/mL (**D–F**) at 3 (**A,D**), 7 (**B,E**) and 14 (**C,F**) days. Osteoblasts not treated were used as control. The ALP concentration was determined using BCIP®/NBT substrate. ALP-stained area/well represents the area of the stain. The asterisk indicates that an ANOVA analysis was performed with Dunnett’s test to compare treatments with the control (*p < 0.05, **p < 0.005, ***p < 0.0005, ****p < 0.0001); a Tukey’s multiple comparison was performed to compare among treatments (letters; a: p < 0.0001; b: p < 0.0005; c: p < 0.005; d: p < 0.05).

The supernatants of the non-biofilm-forming isolates reduced the amount of ALP at all three-time points tested and at both total protein concentrations compared with untreated osteoblasts. The reduction in the amount of ALP was higher in the Se353 isolate than in the HS145 isolate on days 3 and 7 (Fig. 5A–F). The supernatant of strain ATCC1228 did not affect the amount of ALP in osteoblasts except on day 3 at the total protein concentration of 1 µg/mL (Fig. 5D). A greater reduction in the amount of ALP was observed in osteoblasts treated with the supernatants of biofilm-forming isolates at both total protein concentrations, with a greater effect on isolate Se1433 (Fig. 5A–F). The reduction in ALP concentration indicated that the supernatants interfered with the osteogenic differentiation of osteoblasts.

**Figure 6:**
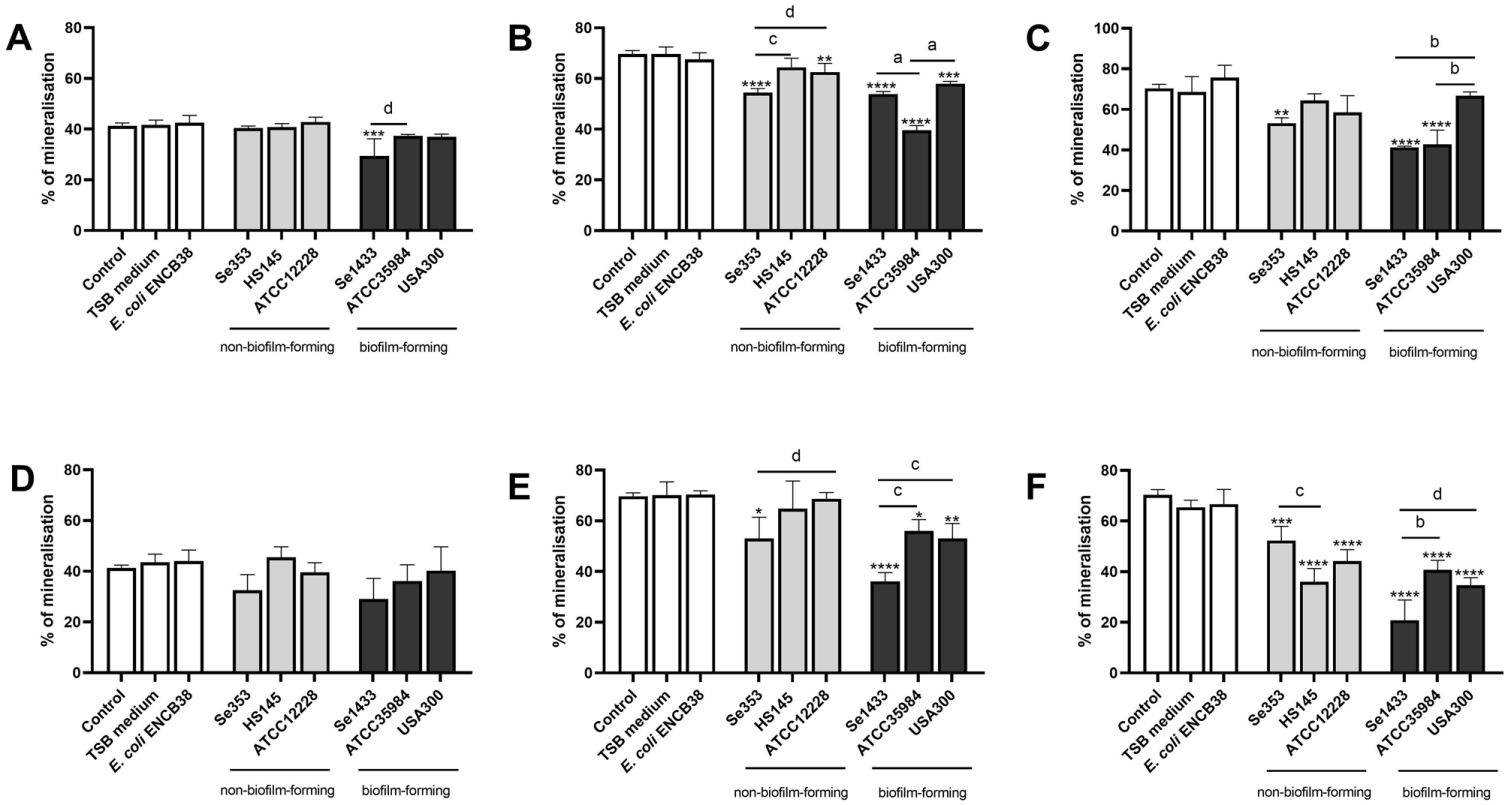
Mineralization percentage of osteoblasts treated with Staphylococcal supernatants. Osteoblasts (cell line MG-63) were grown under osteogenic conditions and treated with supernatant protein concentrations of 0.1 (**A–C**) and 1 µg/mL (**D–F**) at 3 (**A,D**), 7 (**B,E**) and 14 (**C,F**) days. Osteoblasts untreated were used as a control. The mineralization was evaluated by Alizarin red S stain. The mineralization percentage was calculated based on control cells. The asterisk indicates that an ANOVA analysis was performed with Dunnett’s test to compare treatments with the control (*p < 0.05, **p < 0.005, ***p < 0.0005, ****p < 0.0001); a Tukey’s multiple comparison was performed to compare among treatments (letters; a: p < 0.0001; b: p < 0.0005; c: p < 0.005; d: p < 0.05).

Untreated osteoblasts increased the mineralization percentage on days 3 and 7, which was maintained on day 14, indicating that osteoblasts were mineralized in vitro. Supernatants from non-biofilm-forming isolates showed a time-dependent pattern in osteoblast mineralization. At 3 days, these supernatants did not reduce the mineralization percentage (Fig. 6A,D), however at 7 and 14 days the supernatant (both concentrations) from isolate Se353 caused a decrease in mineralization percentage (Fig. 6B,C,E,F) and supernatant from isolate HS145 reduced mineralization percentage only on day 14 at the 1 µg/mL concentration (Fig. 6F), similar effect was true for ATCC12228 strain. The supernatants of the biofilm-forming isolates also showed different behaviors in the reduction of mineralization percentage in osteoblasts; however, isolate Se1433 was more consistent on the various days, except for day 3 at the total concentration of 1 µg/mL (Fig. 6A–F). These phenotypic results showed that the supernatants of Se353 and HS145 delayed the osteogenic process and that the Se1433 supernatant showed the highest inhibitory effect.

### Expression of osteogenic genes by Staphylococcus planktonic cell supernatant

We studied the transcription of genes involved in the regulation of osteogenic differentiation by measuring mRNA expression levels in osteoblasts cultured under osteogenic and non-osteogenic conditions (differentiated and undifferentiated; the ratio of expression under osteogenic and non-osteogenic conditions).

**Figure 7:**
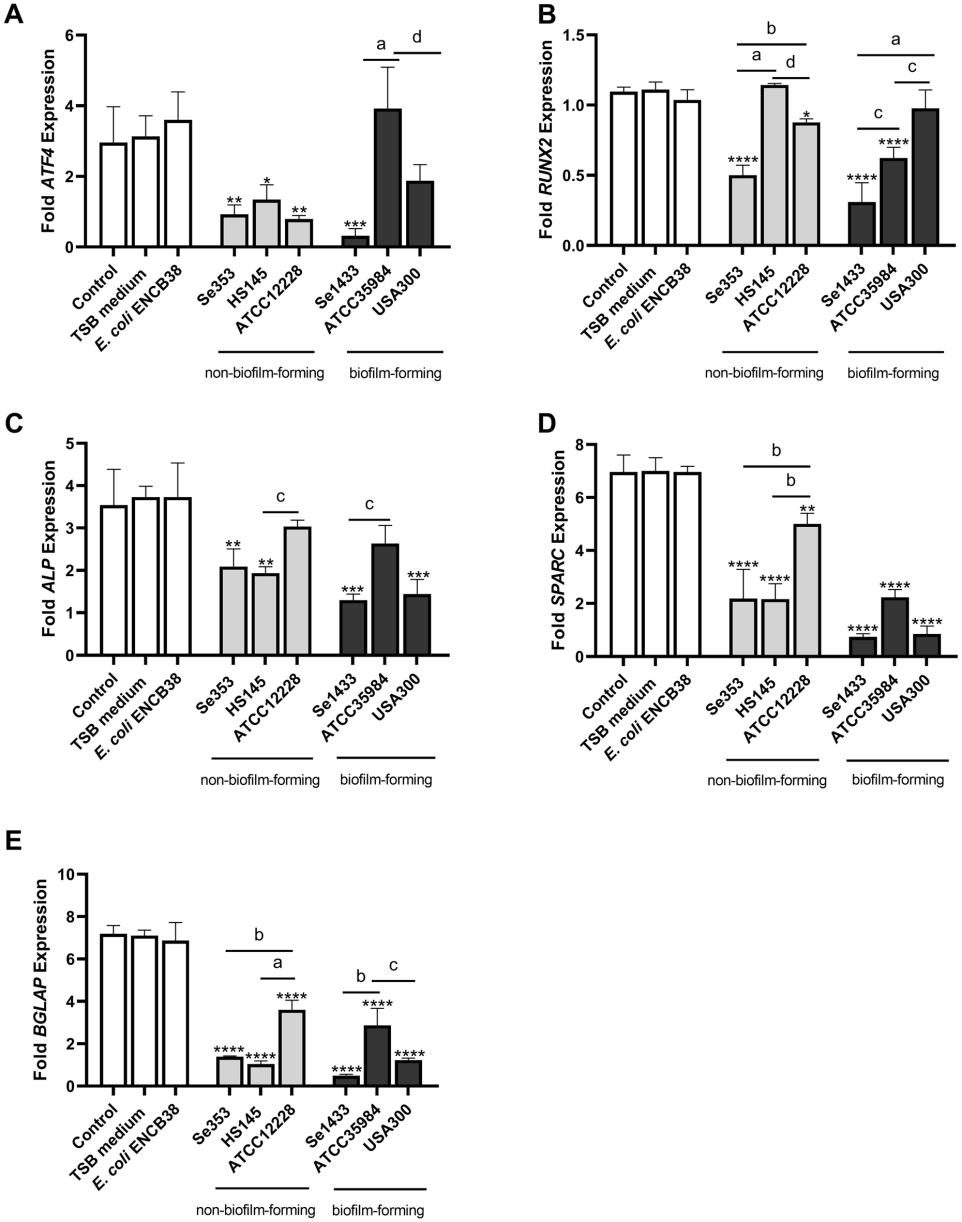
Relative mRNA expression levels of osteoblast differentiation-related genes. Osteoblasts (cell line MG-63) were grown under non-osteogenic and osteogenic conditions and treated with 1 µg/mL de supernatant protein concentration. As control was used osteoblasts grown under non-osteogenic and osteogenic conditions but not treated with supernatants. The mRNA expression was evaluated by RT-qPCR. The expression level was determined by fold of expression of each gene and the ratio between expression in osteogenic conditions and expression in non-osteogenic conditions was calculated. The differentiation genes were ATF4 (**A**), RUNX2 (**B**) evaluated on day 3, and ALP (**C**), SPARC (**D**), and BGLAP (**E**) were evaluated on day 7. The asterisk indicates that an ANOVA analysis was performed with Dunnett’s test to compare treatments with the control (*p < 0.05, **p < 0.005, ***p < 0.0005, ****p < 0.0001); a Tukey’s multiple comparison was performed to compare among treatments (letters; a: p < 0.0001; b: p < 0.0005; c: p < 0.005; d: p < 0.05).

In this study, the transcription factors ATF4 and RUNX2 were analyzed. Supernatants from non-biofilm-forming isolates reduce (threefold) the level of ATF4 expression on day 3 (Fig. 7A). On days 7 and 14, there was a recovery of the level of ATF4 expression in non-biofilm-forming isolates (Supplementary Fig. 2), whereas in supernatants from biofilm-forming isolates, only isolate Se1433 reduced for more than twofold the level of ATF4 expression on the three days assayed (Fig. 7A, Supplementary Fig. 2). Similarly, for the RUNX2 gene, an expression reduction (twofold) in the non-biofilm-forming isolate Se353 on day 3, and the Se1433 isolate showed an expression reduction of more than twofold on the 3 days (Fig. 7B, Supplementary Fig. 2).

ALP gene expression was reduced on days 7 (Fig. 7C) and 14 (Supplementary Fig. 2) in cells treated with supernatants from non-biofilm-forming (1.5-fold) and biofilm-forming (for more than twofold) isolates (Fig. 7C, Supplementary Fig. 2). The expression levels of SPARC and BGLAP decreased by more than twofold on days 3 (Supplementary Fig. 2), 7 (Fig. 7D) and 14 (Supplementary Fig. 2) in both supernatants (non-biofilm-forming and biofilm-forming), and the levels of BGLAP expression were reduced by more than twofold on days 3 (Supplementary Fig. 2), 7 (Fig. 7E) and 14 (Supplementary Fig. 2). These results demonstrate that the expression of these genes, regulated by ATF4 and RUNX2 transcription factors, is decreased by the Se353 and HS145 supernatants; however, the highest effect was observed for the Se1433 supernatant.

### Staphylococcal planktonic cell supernatant increased RANK-L expression

**Figure 8:**
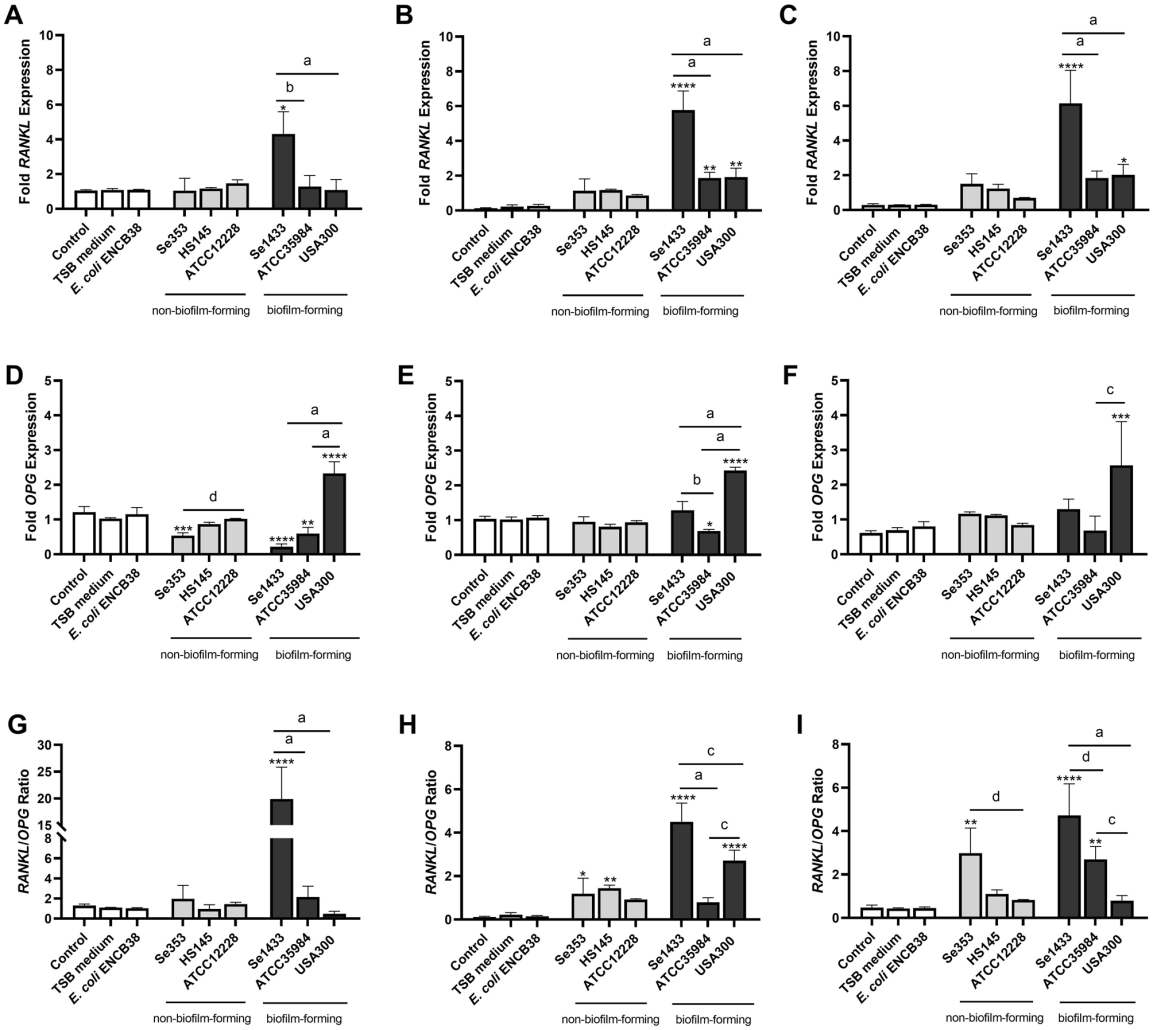
RANK-L and OPG mRNA expression in osteoblasts treated with supernatants. Osteoblasts (cell line MG-63) were grown under non-osteogenic and osteogenic conditions and treated with 1 µg/mL de supernatant protein concentration. Osteoblasts not treated with the supernatant in both growth conditions were used as control. The mRNA expression levels were determined using RT-qPCR. The expression level was determined by fold de expression of each gene, the ratio between expression in osteogenic conditions and expression in non-osteogenic conditions was calculated. The expression level of RANK-L at 3 (**A**), 7 (**B**) and 14 (**C**) days. The expression level of OPG at 3 (**D**), 7 (**E**) and 14 (**F**) days. RANK-L/OPG expression level ratio at 3 (**G**), 7 (**H**) and 14 (**I**) days. The asterisk indicates that an ANOVA analysis was performed with Dunnett’s test to compare treatments with the control (*p < 0.05, **p < 0.005, ***p < 0.0005, ****p < 0.0001); a Tukey’s multiple comparison was performed to compare among treatments (letters; a: p < 0.0001; b: p < 0.0005; c: p < 0.005; d: p < 0.05).

On days 7 and 14, RANK-L expression increased more than fivefold in cells treated with the supernatant from non-biofilm-forming isolates, except for strain ATCC12228, compared to that in control cells (Fig. 8A–C). RANK-L expression increased more than 20-fold in the supernatant from the biofilm-forming Se1433 isolate (Fig. 8A–C). In contrast, the level of OPG gene expression did not decrease in the different supernatants compared to control cells on days 7 and 14, except for the *S. aureus* USA300 supernatant (Fig. 8D–F), with an increase of more than twofold.

The ratio of RANK-L/OPG increased sixfold and tenfold on days 7 and 14, respectively, in osteoblasts treated with the supernatants of the non-biofilm-forming Se353 isolate, and the highest increase in the RANK-L/OPG ratio (39-fold) was observed on day 7 for the biofilm-forming isolate Se1433 (Fig. 8G–I).

## Discussion

Biofilm formation is the main virulence mechanism of *S. epidermidis* compared to *S. aureus*, which contains more virulence elements^41^. Patients with PJI have biofilms in the bone^19^, suggesting that *S. epidermidis* biofilm functions for bacterial protection against the immune system and antibiotic action and induces damage or the development of PJI. *S. epidermidis* biofilms do not fully explain the development of PJI because there are *S. epidermidis* isolates of PJI that do not form biofilm^42^. It is estimated that 30–40% of PJI isolates are not biofilm producers^43,44^, suggesting that these isolates may also alter the biological function of osteoblasts in a biofilm-independent manner. In this study, the supernatant of planktonic *S. epidermidis* cells with a non-biofilm-forming phenotype produced alterations in osteoblast function, such as cell viability, induction of apoptosis, loss of cell differentiation, and RANK-L production; these same changes were also observed in the supernatant of the biofilm-forming isolate Se1433, *S. epidermidis* ATCC35984^38,39^, and *S. aureus* USA300^22^.

The alteration of osteoblast biological function by planktonic cell supernatants from isolates with clinical (Se353) and commensal (HS145) non-biofilm-forming phenotypes indicated that bacteria do not require a biofilm-forming state to alter the function of these cells; however, this ability was better for the biofilm-forming Se1433 supernatant. To our knowledge, this is the first report showing that biofilms are not the only important element in altering osteoblast function, that *S. epidermidis* with a non-biofilm-forming phenotype can also do so, and that this effect does not depend on the source of isolation (clinical or commensal).

The compounds released by bacteria in the biofilm or planktonic state have been poorly studied. In a proteomic study of the supernatants from biofilm of *S. aureus* USA300 that damage the osteoblast functions, the authors propose that some soluble virulence factors such as α-hemolysin, γ-hemolysin, and staphopain B could be related to osteoblast cell apoptosis, and alpha-hemolysin could be an essential role in the pathogenesis of *S. aureus*^22^. Hemolysins (alpha-, beta-, gamma-, and delta-hemolysins) produced by *S. aureus* can damage host cell membranes^45^. The phenol-soluble modulin (PSM) α of the community-acquired methicillin-resistant *Staphylococcus aureus* (CA-MRSA) strain can kill osteoblasts^46^. The modulins of *S. epidermidis* play several roles in the commensal and pathogenic states, including PSMalpha, PSMdelta, delta-toxin, (PSMgamma), PSM-mec, and PSMbeta (PSMbeta1, PSMbeta2). These modulins have been reported to play a role in biofilm formation and evasion of the immune response. In addition, its role in interspecies competition and the cytolytic activities of PSMalpha and PSMdelta have been reported^47^; however, these PSMs are expressed at low concentrations. In contrast, protease SepA is secreted by *S. epidermidis* and contributes to the evasion of *S. epidermidis* to neutrophils activity through the destruction of antimicrobial peptides (AMPs) produced by these immune cells^48^. Comparative serologic proteome analysis of *S. aureus* and *S. epidermidis* exoproteins in PJI identified five exoproteins in *S. aureus* and autolysin E and App (accumulation associated protein) in *S. epidermidis*^49^. The effect of secretome of both species on immune cells also differ, whereas the stimulation of monocyte-derived dendritic cells (moDC) with *S. aureus* secretome induces the production of IFN-ɤ and the proliferation of CD4+ T cells, the *S. epidermidis* secretome induce the IL-10 production in the same cells and the activation of regulatory T cells (Treg)^50^. On the other hand, the clinical isolate of *S. epidermidis* from PJI induced a low IL-1β production in human neutrophils compared with skin isolates, suggesting its ability to evade the innate immune response^51^.

In this study, we obtained the supernatant and bacterial culture medium in a manner different from that used in other studies. To obtain the supernatant, we collected all compounds produced during biofilm formation (24 h) in a 6-well plate without washing; in other reports, a Transwell system and continuous washes were performed^22,38,39^. Despite this change, we observed alterations in osteoblast function at two supernatant total protein concentrations (0.1 and 1 µg/mL), suggesting that at low total protein concentrations (tenfold dilution) there is a biological effect. Considering the culture medium, supernatants were obtained in TSB growth medium, whereas in other studies, DMEM^22^ or DMEM-osteogenic medium^38,39^ was used. We attribute that the production of a bacterial factor that alters osteoblast function is independent of TSB medium since control osteoblasts stimulated with TSB medium (no bacterial growth) and with supernatant from a planktonic culture of *E. coli* (non-biofilm producing), showed no significant changes in osteoblast function, in contrast, supernatants from planktonic *S. epidermidis* cells had a very similar effect on osteoblasts with DMEM medium or DMEM-osteogenic medium already reported^22,38,39^. Furthermore, the alteration in osteoblast biology was *Staphylococcus*-dependent, as the *E. coli* supernatant did not produce alterations.

*S. epidermidis* has an open pan-genome and isolates from different sources have different genotypes and phenotypes^52^. We have reported that *S. epidermidis* isolates from PJI are more resistant to biofilm production under various in vitro biofilm induction conditions and that most commensal isolates do not form biofilms^42^. Furthermore, commensal isolates from healthy skin can induce biofilm formation when exposed to neutrophil proteases, such as cathepsin G, cathepsin B, proteinase-3 and metalloproteinase-9^53^. In this study, we demonstrated that both the clinical isolate Se353 and commensal isolate HS145 affected osteoblast function, suggesting that their influence on osteoblast function is independent of biofilm status, which is surprising because it is well known that commensal isolates are not potentially virulent. The *S. epidermidis* ATCC12228 strain (non-biofilm-forming) did not induce alterations in osteoblast function, which we attribute to the fact that it is a laboratory strain already adapted to laboratory growth conditions; thus, it has lost its environmental origin capacity.

PJI relapse is associated with the ability of *S. epidermidis* to form biofilms and antimicrobial resistance^54^. Moreover, antimicrobial treatment failure in PJI correlates with the resistance profile and presence of genetic resistance elements in the genomes of *S. epidermidis* and *S. aureus*^55^. Furthermore, the genetic resistance elements to β-lactams, aminoglycosides and chlorhexidine, present in *S. epidermidis* from PJIs patients are different from nasal isolates^56^, which suggests that it is necessary to know the antimicrobial resistance profile before the treatment. The mechanism proposal for infection by *S. epidermidis* biofilm is mainly by persistence or dormant cells^57–59^. This process involves the adhesion of *S. epidermidis*^60^ to the medical implant introduced into the tissue, which is thought to change its metabolic state to dormancy^61,62^. In this state, there is tolerance to antibiotics, in which the bacteria persist and adapt to environmental conditions, and bacteria in a latent state have low immunogenic reactivity^63^. One evidence of this metabolic change is in the process of acute to chronic bone infection where it has been observed that the bacterium becomes established in a less virulent form (so-called “small colony variant”), with characteristics of increased intracellular persistence, antibiotic resistance, and reduced induction of cytokine release and stimulation of the immune system^64,65^.

In contrast, the transcription factor RUNX2 is involved in the early osteoblast differentiation pathway, specifically during the passage of osteochondroprogenitor cells. The transcription factor ATF4 is also involved in pre-osteoblast differentiation. Both factors are master regulators of osteoblast differentiation and play an important role in cell-fate decisions from mesenchymal stem cells (MSCs)^66,67^. Supernatant from non-biofilm-forming isolates reduced RUNX2 and ATF4 mRNA expression by more than twofold compared to control, suggesting that the factor released by *S. epidermidis* regulates osteoblast differentiation process. This low expression of RUNX2 and ATF4 alters the expression of other differentiation genes regulated by them, as is the case of alkaline phosphatase (ALP) which is expressed early and has the function of precipitating calcium phosphate in bone, or osteonectin (SPARC) which binds calcium and initiates mineralization and promotes the formation of mineral crystals, and finally, osteocalcin (BGLAP), a hormone that is incorporated into the bone matrix and helps calcium fixation. The expression of these differentiation genes correlated with the phenotypic data of ALP activity and cell mineralization, suggesting that the supernatant also affected late differentiation, such as the mineralization of the extracellular matrix produced by osteoblasts.

Osteoblasts in the presence of pathogenic bacteria produce several effects such as cytokine release, RANK-L production (to maintain osteoclastic activity), downregulation of the decoy receptor OPG, misproduction of bone matrix and mineralization, and finally, osteoblast death^68,69^. All of these events contribute to bone loss. RANK-L expression was fivefold higher than that in the control at day 7-day time point, indicating that *S. epidermidis* supernatant induced early RANK-L production in osteoblasts. RANK-L acts on osteoclast differentiation during bone resorption. RANK-L is produced in osteoblasts stimulated with *S. epidermidis*^38,39^. Moreover, the RANK-L/OPG ratio is considered a strong predictor of rapid and persistent bone loss in rheumatoid arthritis, osteoporosis, and periodontal disease^35,70^, making it an indicator of osteoclast differentiation^5,6^. Osteoblasts stimulated with the studied supernatants showed a sixfold increase in the RANK-L/OPG ratio, indicating that the osteoclastic process occurred because the antagonist of this process, OPG, was not modified. Thus, it can be stated that the supernatant of *S. epidermidis* planktonic cells and the biofilm state, are inducers of bone resorption through the production of RANK-L by osteoblasts.

The other effects of *S. epidermidis* on osteoblasts have also been studied. For example, it has been established that osteoblasts aid in the biofilm formation of *S. epidermidis* on medical devices, as the lack of osteoblasts causes *S. epidermidis* not to form biofilms^71^. In contrast, in a murine model of implant-associated osteomyelitis caused by *S. epidermidis* ATCC35984, the inhibition of bone integration by the biofilm formed on the implant was observed without osteolysis and with a low degree of inflammation^38^. *S. epidermidis* can invade osteoblasts and persist in intracellular compartments, protecting itself from antimicrobial activity^65,72^. The serine-aspartate repeat protein of *S. epidermidis* binds to alpha integrin V beta3 in osteoblasts to initiate osteomyelitis^73^. *S. epidermidis* induces the expression of inflammatory cytokines (IL-6 and IL-8) in osteoblasts, as well as biofilm components such as exopolysaccharides (EPS), including lipoteichoic acid and the heat shock protein GroEL, which contribute to the persistence of the inflammatory response associated with implant infections^74^. This demonstrates that *S. epidermidis* can affect the bone and cause damage, although to a lesser degree than *S. aureus*.

In conclusion, we showed that the supernatants of *S. epidermidis* isolates from PJI and healthy skin (commensal) with a non-biofilm-forming phenotype alter the function of osteoblasts (induction of apoptosis, failure of cell differentiation, and activation of osteoblasts and bone resorption) similar to that of *S. aureus* USA300 and *S. epidermidis* ATCC35984 (biofilm-forming), suggesting that biofilm status contributes to impaired osteoblast function, and that the planktonic state can do so independently of biofilm production.

## Materials and methods

### Cell line and culture conditions

Human osteoblast MG-63 cell line (CRL-1427TM) was obtained from the American Type Culture Collection (ATCC Manassas, VA, USA). The MG-63 cells in passage 12 were propagated in 25 cm^2^ cell culture flasks at 37 °C, 5% CO_2_ atmosphere with α-MEM medium (HyClone, GE Healthcare Life Sciences, Logan, Utah, USA) supplemented with 10% fetal bovine serum (FBS) (Gibco, Thermo Fisher Scientific, Waltham, MA, USA), penicillin 100 U/mL and streptomycin at 100 mg/mL. In some conditions the maintaining medium was added of ascorbic acid (50 μM) and β-glycerophosphate (20 mM) (Sigma, St. Louis, MO, USA) for osteogenic differentiation.

### Isolates and strains

Two isolates of *S. epidermidis* from patients with prosthetic joint infection (PJI) were used: one with a biofilm-forming phenotype (Se1433) and the other with a non-biofilm-forming phenotype (Se353). One isolate of *S. epidermidis* from the skin of a healthy subject (HS145) with a non-biofilm-forming phenotype was included. All clinical and commensal *S. epidermidis* isolates were the same as those used by our group and published previously^40^. Therefore, patients and healthy humans were not included in the study. *S. epidermidis* strain RP62A with a biofilm-forming phenotype (ATCC 35984) and *S. epidermidis* strain ATCC12228 with a non-biofilm-forming phenotype were also used. *Staphylococcus aureus* strain USA-300, with biofilm-forming and methicillin-resistant phenotypes, was included as a positive control for its damaging effect on osteoblast function. *Escherichia coli* ENCB38 isolated from human feces was used as a non-Staphylococcal control to establish whether the effect of the supernatants on osteoblasts was specific to the *Staphylococcus* genus.

### Obtaining bacterial supernatants for osteoblast MG-63 cells stimulation assays

An overnight bacterial culture was diluted 1:200 and placed in a 6-well plate (Nest Scientific Inc., NJ, USA) in tryptic soy broth (TSB; BD Difco, Heidelberg, Germany). The plate was incubated at 37 °C without shaking for 24 h, the supernatant was recovered and filtered with a 0.2 μm filter (EMD Millipore, Billerica, MA, USA). The pH was adjusted to 7.2 and stored at − 72 °C until use. Supernatants were checked for sterility using plate assays with culture medium and in the absence of bacteria. The total protein concentration in all supernatants was determined using the Lowry method^75^, and subsequent experiments were based on the protein concentration.

### Cytotoxicity assay of supernatants in osteoblast MG-63 cells

This assay was performed using MG-63 osteoblasts. Confluent cell monolayers were prepared in 24-well plates (Nest Scientific, Inc.) at 2 × 10^5^ cells per well. The supernatant concentrations (0.01, 0.1, 1, and 2 µg/mL of total protein) were added to the cells. Ursolic acid (1 mg/mL) (Merck, Darmstadt, Germany) was used as a cytotoxicity control. Incubation was maintained for 24 h, after which the culture medium was discarded, and the cells were washed with 1× PBS. Then, 500 µL MTT (3-(4,5-dimethylthiazol-2-yl)-2,5-diphenyl-tetrazolium bromide; Sigma) (5 mg/mL) was added, and the plates were incubated under the same conditions mentioned above for 3 h. MTT was discarded and 500 µL of DMSO (Merck) was added to dissolve the formazan crystals. The plates were read at 570 nm using a Multiskan GO spectrophotometer (Thermo Fisher Scientific).

### Cell viability and proliferation of osteoblast MG-63 cells treated by bacterial supernatants

The viability of osteoblast MG-63 cells was evaluated using a vital stain LIVE/DEAD© assay kit (Invitrogen, Waltham, MA, USA). For that, a monolayer of osteoblasts were prepared in a 24-well plate (Nest Scientific Inc.) with 2 × 10^5^ cells in each well, supernatant (0.1 or 1 µg/mL of total protein) was added and incubated for 48 h. Then, the medium was removed, and cells were washed with PBS 1x. Staining was performed according to manufacturer’s instructions. Briefly, cells were incubated with fluorochromes (Calcein, AM, and EthD-1) from the kit for 15 min. The monolayer was analyzed using a fluorescence microscope (Nikon Eclipse E800; Melville, NY, USA). The images were obtained with the software ACT-1 (Nikon) and processed using the software ImageJ® (NIH; http://rsb.info.nih.gov/ij).

The total cellular DNA was quantified using the CyQUANT® cell proliferation assay (Molecular Probes, Eugene, OR, USA) following the manufacturer’s recommendations. Monolayers of osteoblasts were prepared in 96 wells/plate with 5 × 10^4^ cells/well and treated with supernatants (0.1 or 1 µg/mL of total protein) for 3, 7, and 14 days. Then, 50 µL of colorant solution was added after 5 min. A standard curve of 10–1000 ng/mL DNA was generated for this experiment. The absorbance was read at an excitation wavelength of 485 nm and an emission wavelength of 538 nm using a Multiskan GO spectrophotometer (Thermo Fisher Scientific).

### Apoptosis assay of osteoblast MG-63 cells treated by bacterial supernatants

Osteoblast MG-63 cells were prepared in a 24-well plate (Nest Scientific Inc.) with 2 × 10^5^ cells in each well, and supernatant (0.1 or 1 µg/mL of total protein) was added. Osteoblast apoptosis was assessed after 48 h of incubation with the supernatants. An Annexin V-FITC apoptosis detection kit (BD, Franklin Lakes, NJ, USA) was used for fluorescence-activated cell sorting (FACS) using a FACSCalibur flow cytometer (BD). The data were analyzed using FlowJo software (Tree Star, Inc., Ashland, OR, USA). Cisplatin 50 µM (CDDP; Sigma) was used as a positive control of apoptosis.

### Alkaline phosphatase measurement of osteoblast MG-63 cells

Alkaline phosphatase activity was determined using a biochemical colorimetric test. The assay uses BCIP®/NBT (Sigma) substrate. Briefly, a cell monolayer of 2 × 10^5^ cells was cultured in 24-well plates (Nest Scientific Inc.) under osteogenic conditions (ascorbic acid 50 μM and β-glycerophosphate 20 mM; Sigma) and non-osteogenic conditions (only growth medium) for up to 14 days, the supernatant concentrations of 0.1 and 1 µg/mL of total protein were added to cells in both conditions. After stimulation, the cells were washed thrice with PBS 1× and fixed with 4% paraformaldehyde (PFA; Sigma) for 15 min at room temperature. Immediately, 500 µL of BCIP®/NBT substrate was added and incubated for 1 h. The reaction was terminated by washing the cells with distilled water. Phosphatase hydrolyses the BCIP substrate in the presence of NBT, forming a dark blue color. Photographs were taken from three wells for each treatment and processed into RGB colors, and color intensity was calculated using ImageJ software^76^. Color intensity was assumed to be proportional to the phosphatase activity. The threshold was adjusted to eliminate non-specific pixels. Finally, the area values, known as the percentage staining area of each well, were obtained.

### Alizarin red S stain of osteoblast MG-63 cells

Intracellular calcium deposition in osteoblasts was assessed by Alizarin Red S staining. Osteoblasts were cultured in 24-well plates (Nest Scientific Inc.) under osteogenic conditions until they reached 80% confluence. Subsequently, supernatants were added at a concentration of 0.1 and 1 µg/mL of total protein, with replacement every second day for up to 14 days. The wells were then washed three times with 1× PBS (no calcium or magnesium), fixed with 4% PFA for 15 min, and stained with 40 mM alizarin red S (Sigma) for 30 min. The plate was washed with 1× PBS, 10% acetic acid (Sigma) was added, and the plate was shaken constantly for 30 min in the dark. The cell monolayer was scraped off and transferred to a vial.

The samples were heated at 85 °C for 10 min and immediately placed on ice for 5 min. The samples were centrifuged at 10,000 rpm for 15 min, then 200 µL of the supernatants were transferred to another tube and 75 µL of 10% ammonium hydroxide (Sigma) was added^77^. Finally, samples were transferred to 96-well plates. Simultaneously, an alizarin red S (0–40 mM) standard curve was generated. The absorbance was read at 405 nm using a Multiskan GO spectrophotometer (Thermo Fisher Scientific).

### RNA extraction, synthesis of complementary DNA, and PCRs of osteoblast MG-63 cells treated with bacterial supernatants

Osteoblast MG-63 of 1 × 10^6^ cells were cultured in 6-well plates (Nest Scientific Inc.) with α-MEM basal medium or supplemented with ascorbic acid (50 μM) and β-glycerophosphate (20 mM). Then, they were stimulated with the supernatants of each *S. epidermidis,* or *S. aureus* isolates separately, and the medium and supernatant were changed every 2 days for stimulation times of 3, 7, and 14 days. After each incubation time, the culture medium was removed. Then 1 mL of TRIsure® (Bioline, London, UK) was added to each well, and the lysates were stored in 1.5 mL vial at − 72 °C. RNA purification was performed following the methodology described by the manufacturer (Bioline).

For the reverse transcription (RT) reaction, the SuperScript II kit (Invitrogen) was used according to the manufacturer’s instructions. Primers for the expression of genes of interest, *Atf4, Runx2, Alp, Sparc, Bglap, Rank-L*, and *Opg* (osteoblast differentiation-related genes), and *Gapdh* as housekeeping were the same as those of Ref.^22^. Relative expression was determined by the 2^−ΔΔCt^ method. The relative gene expression of each treatment under osteogenic conditions was normalized to that under non-osteogenic conditions.

### Statistical analysis

Data were statistically analyzed using GraphPad software version 8.0. One-way ANOVA with Dunnett’s post hoc test was used to compare the samples with the control (*p < 0.05, **p < 0.005, ***p < 0.0005, ****p < 0.0001), and Tukey’s post hoc test was used to compare all experimental conditions (letters; a: p < 0.0001; b: p < 0.0005; c: p < 0.005; d: p < 0.05). The p < 0.05 values were considered statistically significant.

### Supplementary Information

Supplementary Figures.

## Data Availability

The datasets generated in this study are available from the corresponding author upon request.

## References

[1] Chotiyarnwong P, McCloskey EV (2020). Pathogenesis of glucocorticoid-induced osteoporosis and options for treatment. Nat. Rev. Endocrinol..

[2] Kong YY (1999). OPGL is a key regulator of osteoclastogenesis, lymphocyte development and lymph-node organogenesis. Nature.

[3] Suda T (1999). Modulation of osteoclast differentiation and function by the new members of the tumor necrosis factor receptor and ligand families. Endocr. Rev..

[4] Yasuda H (1998). Identity of osteoclastogenesis inhibitory factor (OCIF) and osteoprotegerin (OPG): A mechanism by which OPG/OCIF inhibits osteoclastogenesis in vitro. Endocrinology.

[5] Marriott I (2004). Osteoblast responses to bacterial pathogens. Immunol. Res..

[6] Montanaro L (2011). Scenery of *Staphylococcus* implant infections in orthopedics. Future Microbiol..

[7] Ong KL (2009). Prosthetic joint infection risk after total hip arthroplasty in the medicare population. J. Arthroplasty.

[8] Kurtz SM (2010). Prosthetic joint infection risk after TKA in the medicare population. Clin. Orthop. Relat. Res..

[9] Davis JS (2022). Predictors of treatment success after periprosthetic joint infection: 24-month follow up from a multicenter prospective observational cohort study of 653 patients. Open Forum Infect. Dis..

[10] Gatti M (2022). Orthopaedic implant-associated staphylococcal infections: A critical reappraisal of unmet clinical needs associated with the implementation of the best antibiotic choice. Antibiotics.

[11] Triffault-Fillit C (2019). Microbiologic epidemiology depending on time to occurrence of prosthetic joint infection: A prospective cohort study. Clin. Microbiol. Infect..

[12] Costerton JW, Stewart PS, Greenberg EP (1999). Bacterial biofilms: A common cause of persistent infections. Science.

[13] Costerton JW, Montanaro L, Arciola CR (2005). Biofilm in implant infections: Its production and regulation. Int. J. Artif. Organs.

[14] Costerton JW (1999). Introduction to biofilm. Int. J. Antimicrob. Agents.

[15] Hall-Stoodley L, Costerton JW, Stoodley P (2004). Bacterial biofilms: From the natural environment to infectious diseases. Nat. Rev. Microbiol..

[16] Brady RA, Leid JG, Calhoun JH, Costerton JW, Shirtliff ME (2008). Osteomyelitis and the role of biofilms in chronic infection. FEMS Immunol. Med. Microbiol..

[17] Marrie TJ, Costerton JW (1985). Mode of growth of bacterial pathogens in chronic polymicrobial human osteomyelitis. J. Clin. Microbiol..

[18] Sedghizadeh PP (2009). Microbial biofilms in osteomyelitis of the jaw and osteonecrosis of the jaw secondary to bisphosphonate therapy. J. Am. Dent. Assoc..

[19] Dastgheyb SS (2015). Staphylococcal persistence due to biofilm formation in synovial fluid containing prophylactic cefazolin. Antimicrob. Agents Chemother..

[20] Esteban J (2010). Biofilm development by clinical isolates of *Staphylococcus* spp. from retrieved orthopedic prostheses. Acta Orthop..

[21] O’Neill E (2007). Association between methicillin susceptibility and biofilm regulation in *Staphylococcus aureus* isolates from device-related infections. J. Clin. Microbiol..

[22] Sánchez CJ (2013). *Staphylococcus aureus* biofilms decrease osteoblast viability, inhibits osteogenic differentiation, and increases bone resorption in vitro. BMC Musculoskelet. Disord..

[23] Buxton TB, Horner J, Hinton A, Rissing JP (1987). In vivo glycocalyx expression by *Staphylococcus aureus* phage type 52/52A/80 in *S. aureus* osteomyelitis. J. Infect. Dis..

[24] Morgenstern M (2016). Biofilm formation increases treatment failure in *Staphylococcus epidermidis* device-related osteomyelitis of the lower extremity in human patients. J. Orthop. Res..

[25] Stewart PS (2015). Antimicrobial tolerance in biofilms. Microbiol. Spectr..

[26] Morawietz L (2006). Proposal for a histopathological consensus classification of the periprosthetic interface membrane. J. Clin. Pathol..

[27] Yokota K (2014). Combination of tumor necrosis factor α and interleukin-6 induces mouse osteoclast-like cells with bone resorption activity both in vitro and in vivo. Arthritis Rheumatol..

[28] Kwan Tat S, Padrines M, Théoleyre S, Heymann D, Fortun Y (2004). IL-6, RANKL, TNF-alpha/IL-1: Interrelations in bone resorption pathophysiology. Cytokine Growth Factor Rev..

[29] Chakravarti A, Raquil M-A, Tessier P, Poubelle PE (2009). Surface RANKL of toll-like receptor 4-stimulated human neutrophils activates osteoclastic bone resorption. Blood.

[30] Gaida MM (2012). Polymorphonuclear neutrophils in osteomyelitis: Link to osteoclast generation and bone resorption. Eur. J. Inflamm..

[31] Dapunt U, Hänsch GM, Arciola CR (2016). Innate immune response in implant-associated infections: Neutrophils against biofilms. Materials.

[32] Heim CE (2018). Human prosthetic joint infections are associated with myeloid-derived suppressor cells (MDSCs): Implications for infection persistence. J. Orthop. Res..

[33] Heim CE, Vidlak D, Kielian T (2015). Interleukin-10 production by myeloid-derived suppressor cells contributes to bacterial persistence during *Staphylococcus aureus* orthopedic biofilm infection. J. Leukoc. Biol..

[34] Guenther F, Stroh P, Wagner C, Obst U, Hänsch GM (2009). Phagocytosis of staphylococci biofilms by polymorphonuclear neutrophils: *S. aureus* and *S. epidermidis* differ with regard to their susceptibility towards the host defense. Int. J. Artif. Organs.

[35] Boyce BF, Xing L (2008). Functions of RANKL/RANK/OPG in bone modeling and remodeling. Arch. Biochem. Biophys..

[36] Claro T (2011). *Staphylococcus aureus* protein A binds to osteoblasts and triggers signals that weaken bone in osteomyelitis. PLoS ONE.

[37] Widaa A, Claro T, Foster TJ, O’Brien FJ, Kerrigan SW (2012). *Staphylococcus aureus* protein A plays a critical role in mediating bone destruction and bone loss in osteomyelitis. PLoS ONE.

[38] Tomizawa T (2020). Biofilm producing *Staphylococcus epidermidis* (RP62A strain) inhibits osseous integration without osteolysis and histopathology in a murine septic implant model. J. Orthop. Res..

[39] Tübel J (2021). Patient-specific effects of soluble factors from *Staphylococcus aureus* and *Staphylococcus epidermidis* biofilms on osteogenic differentiation of primary human osteoblasts. Sci. Rep..

[40] Ortega-Peña S (2019). *sesA*, *sesB*, *sesC*, *sesD*, *sesE*, *sesG*, *sesH*, and *embp* genes are genetic markers that differentiate commensal isolates of *Staphylococcus epidermidis* from isolates that cause prosthetic joint infection. Infect. Dis..

[41] Fey PD, Olson ME (2010). Current concepts in biofilm formation of *Staphylococcus epidermidis*. Future Microbiol..

[42] Martínez-García S (2019). Non-biofilm-forming commensal *Staphylococcus epidermidis* isolates produce biofilm in the presence of trypsin. MicrobiologyOpen.

[43] Hellmark B, Söderquist B, Unemo M, Nilsdotter-Augustinsson Å (2013). Comparison of *Staphylococcus epidermidis* isolated from prosthetic joint infections and commensal isolates in regard to antibiotic susceptibility, *agr* type, biofilm production, and epidemiology. Int. J. Med. Microbiol..

[44] Juárez-Verdayes MA (2013). *Staphylococcus epidermidis* with the *icaA*−/*icaD*−/*IS256*− genotype and protein or protein/extracellular-DNA biofilm is frequent in ocular infections. J. Med. Microbiol..

[45] DeLeo FR, Diep BA, Otto M (2009). Host defense and pathogenesis in *Staphylococcus aureus* infections. Infect. Dis. Clin. N. Am..

[46] Rasigade JP (2013). PSMs of hypervirulent *Staphylococcus aureus* act as intracellular toxins that kill infected osteoblasts. PLoS ONE.

[47] Cheung GY, Joo HS, Chatterjee SS, Otto M (2014). Phenol—Soluble modulins critical determinants of staphylococcal virulence. FEMS Microbiol. Rev..

[48] Cheung GY (2010). *Staphylococcus epidermidis* strategies to avoid killing by human neutrophils. PLoS Pathog..

[49] Nuyttens H, Thomas D, Rogé J, Mignon K (2010). Analyse immunoprotéomique comparative des sécrétomes de souches de *Staphylococcus aureus* et *S. epidermidis* chez les patients souffrant d’infections sur prothèses articulaires (Comparative serologic proteome analysis of *Staphylococcus aureus* and *S. epidermidis* exoproteins in prosthetic joint infections). Pathol. Biol..

[50] Laborel-Préneron E (2015). Effects of the *Staphylococcus aureus* and *Staphylococcus epidermidis* secretomes isolated from the skin microbiota of atopic children on CD4+ T cell activation. PLoS ONE.

[51] Månsson E, Söderquist B, Nilsdotter-Augustinsson Å, Särndahl E, Demirel I (2018). *Staphylococcus epidermidis* from prosthetic joint infections induces lower IL-1β release from human neutrophils than isolates from normal flora. APMIS.

[52] Conlan S (2012). *Staphylococcus epidermidis* pan-genome sequence analysis reveals diversity of skin commensal and hospital infection-associated isolates. Genome Biol..

[53] Gómez-Alonso IS (2022). Low concentration of the neutrophil proteases cathepsin G, cathepsin B, proteinase-3 and metalloproteinase-9 induce biofilm formation in non-biofilm-forming *Staphylococcus epidermidis* isolates. Int. J. Mol. Sci..

[54] Svensson-Malchau K (2021). Biofilm properties in relation to treatment outcome in patients with first-time periprosthetic hip or knee joint infection. J. Orthop. Transl..

[55] Trobos M (2022). Genomics of *Staphylococcus aureus* and *Staphylococcus epidermidis* from periprosthetic joint infections and correlation to clinical outcome. Microbiol. Spectr..

[56] Månsson E, Bech-Johannesen T, Nilsdotter-Augustinsson Å, Söderquist B, Stegger M (2021). Comparative genomics of *Staphylococcus epidermidis* from prosthetic-joint infections and nares highlights genetic traits associated with antimicrobial resistance, not virulence. Microb. Genom..

[57] Bellou V (2022). Persistent coagulase-negative staphylococcal bacteremia in neonates: Clinical, microbiological characteristics and changes within a decade. Antibiotics.

[58] Oliveira F (2022). Involvement of the iron-regulated loci *hts* and *fhuC* in biofilm formation and survival of *Staphylococcus epidermidis* within the Host. Microbiol. Spectr..

[59] Pintens V (2008). The role of *σB* in persistence of *Staphylococcus epidermidis* foreign body infection. Microbiology.

[60] Tang H (2006). Effect of surface proteins on *Staphylococcus epidermidis* adhesion and colonization on silicone. Colloids Surf. B.

[61] Carvalhais V, Cerveira F, Vilanova M, Cerca N, Vitorino R (2015). An immunoproteomic approach for characterization of dormancy within *Staphylococcus epidermidis* biofilms. Mol. Immunol..

[62] Cerca F (2011). *Staphylococcus epidermidis* biofilms with higher proportions of dormant bacteria induce a lower activation of murine macrophages. J. Med. Microbiol..

[63] Cerca F (2014). Dormant bacteria within *Staphylococcus epidermidis* biofilms have low inflammatory properties and maintain tolerance to vancomycin and penicillin after entering planktonic growth. J. Med. Microbiol..

[64] Bogut A (2014). Characterization of *Staphylococcus epidermidis* and *Staphyloccocus warneri* small-colony variants associated with prosthetic-joint infections. J. Med. Microbiol..

[65] Perez K, Patel R (2017). *Staphylococcus epidermidis* small-colony variants are induced by low pH and their frequency reduced by lysosomal alkalinization. J. Infect. Dis..

[66] Lian JB (2004). Regulatory controls for osteoblast growth and differentiation: Role of *Runx/Cbfa/AML* factors. Crit. Rev. Eukaryot. Gene Expr..

[67] Yang X (2004). *ATF4* is a substrate of RSK2 and an essential regulator of osteoblast biology: Implication for Coffin–Lowry syndrome. Cell.

[68] Reott MA, Ritchie-Miller SL, Anguita J, Hudson MC (2008). TRAIL expression is induced in both osteoblasts containing intracellular *Staphylococcus aureus* and uninfected osteoblasts in infected cultures. FEMS Microbiol. Lett..

[69] Young AB, Cooley ID, Chauhan VS, Marriott I (2011). Causative agents of osteomyelitis induce death domain-containing TNF-related apoptosis-inducing ligand receptor expression on osteoblasts. Bone.

[70] Hofbauer L, Kühne C, Viereck V (2004). The OPG/RANKL/RANK system in metabolic bone diseases. J. Musculoskelet. Neuronal Interact..

[71] Zaatreh S (2016). Co-culture of *S. epidermidis* and human osteoblasts on implant surfaces: An advanced in vitro model for implant-associated infections. PLoS ONE.

[72] Valour F (2013). *Staphylococcus epidermidis* in orthopedic device infections: The role of bacterial internalization in human osteoblasts and biofilm formation. PLoS ONE.

[73] Claro T, Kavanagh N, Foster TJ, O’Brien FJ, Kerrigan SW (2015). *Staphylococcus epidermidis* serine–aspartate repeat protein G (SdrG) binds to osteoblast integrin alpha V beta 3. Microbes Infect..

[74] Dapunt U, Giese T, Stegmaier S, Moghaddam A, Hänsch GM (2016). The osteoblast as an inflammatory cell: Production of cytokines in response to bacteria and components of bacterial biofilms. BMC Musculoskelet. Disord..

[75] Lowry OH, Rosbrough NJ, Farr AL, Randall RJ (1951). Protein measurement with the folin phenol reagent. J. Biol. Chem..

[76] Schneider CA, Rasband WS, Eliceiri KW (2012). NIH image to ImageJ: 25 years of image analysis. Nat. Methods.

[77] Stanford CM, Jacobson PA, Eanes ED, Lembke LA, Midura RJ (1995). Rapidly forming apatitic mineral in an osteoblastic cell line (UMR 106–01 BSP). J. Biol. Chem..

