# Supplementary material for: Non-biofilm-forming Staphylococcus epidermidis planktonic cell supernatant induces alterations in osteoblast biological function
Source: Sci Rep. 2024 Jan 20;14:1807. doi: 10.1038/s41598-024-51899-7 (PMC10799936; doi:10.1038/s41598-024-51899-7)
Supplement: Supplementary file 1 — Supplementary Figures. [file 41598_2024_51899_MOESM1_ESM.pdf]

Non-biofilm-forming *Staphylococcus epidermidis* planktonic cell supernatant induces alterations in osteoblast biological function.

Itzia Sidney Gómez-Alonso[a], Gabriel Betanzos-Cabrera[b], Martha Cecilia Moreno-Lafont[c], Mario E. Cancino-Diaz[c], Blanca Estela García Pérez[a], Juan Carlos Cancino-Diaz[a**].

[a]Departamento de Microbiología, Escuela Nacional de Ciencias Biológicas, Instituto Politécnico Nacional, Manuel Carpio, Plutarco Elías Calles, Miguel Hidalgo, 11350, Ciudad de México, México.

[b]Área Académica de Nutrición y Medicina, Instituto de Ciencias de la Salud, Universidad Autónoma del Estado de Hidalgo, Carretera Pachuca-Actopan camino a Tilcuautla s/n., Pueblo San Juan Tilcuautla, 42160, Pachuca Hidalgo, México.

[c]Departamento de Inmunología, Escuela Nacional de Ciencias Biológicas, Instituto Politécnico Nacional, Manuel Carpio, Plutarco Elías Calles, Miguel Hidalgo, 11350, Ciudad de México, México

** Corresponding author: jccancinodiaz@hotmail.com (Juan Carlos Cancino-Diaz).

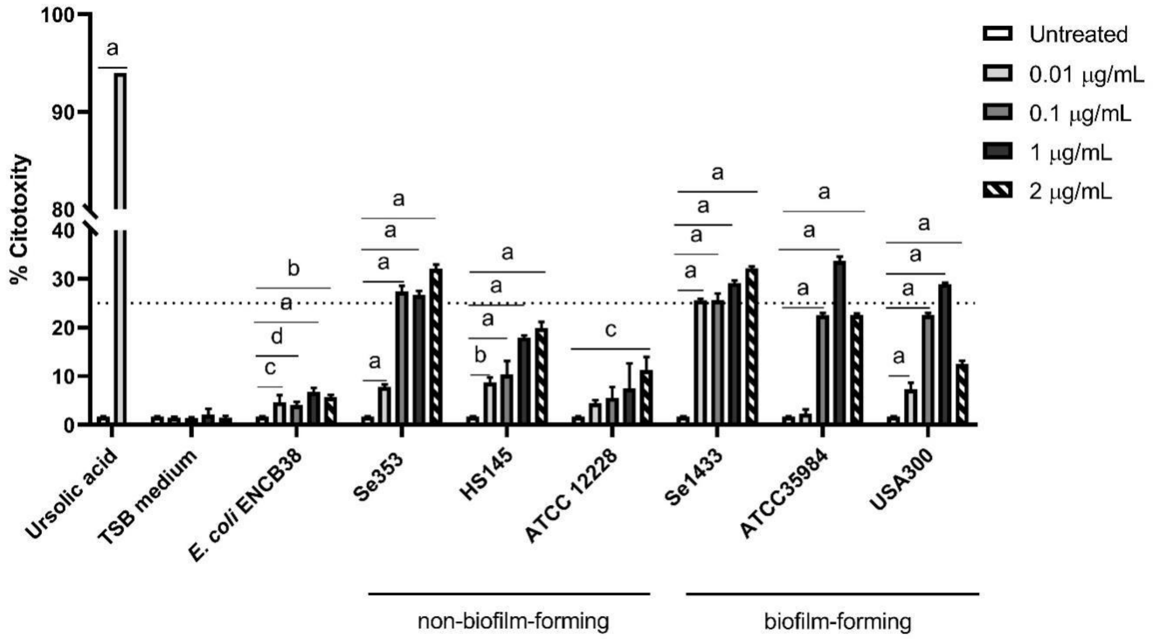

Supplementary figure 1. cytotoxicity of supernatants of non-biofilm-forming and biofilm-forming strains on osteoblasts. Supernatant protein concentrations of 0.01 - 2 were added to the cells for 24 h. Ursolic acid was used as a cytotoxicity control. The asterisk indicates that an ANOVA analysis was performed with Dunnett's test to compare treatments with the control (*= p<0.05, **= p<0.005, ***= p<0.0005, ****= p<0.0001); a Tukey´s multiple comparison was performed to compare among treatments (letters; a: p<0.0001; b: p<0.0005; c: p<0.005; d: p<0.05).

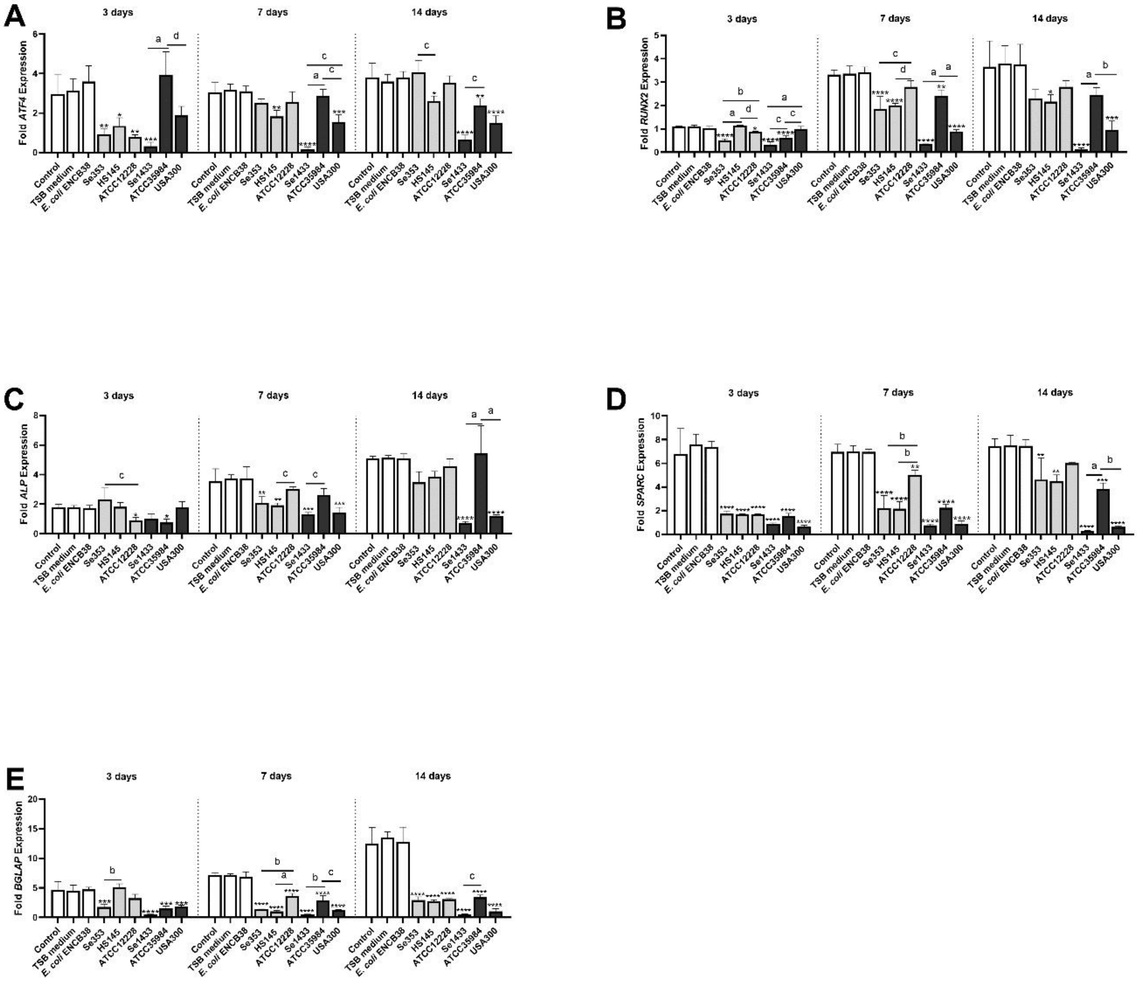

Supplementary figure 2. Relative mRNA expression levels of osteoblast differentiation-related genes. Osteoblasts (cell line MG-63) were grown under non-osteogenic and osteogenic conditions and treated with 1 µg/mL de supernatant protein concentration. As control was used osteoblasts grown under non-osteogenic and osteogenic conditions but not treated with supernatants. The mRNA expression was evaluated by RT-qPCR. The expression level was determined by fold of expression of each gene and the ratio between expression in osteogenic conditions and expression in non-osteogenic conditions was calculated. The asterisk indicates that an ANOVA analysis was performed with Dunnett's test to compare treatments with the control (*= p<0.05, **= p<0.005, ***= p<0.0005, ****= p<0.0001); a Tukey´s multiple comparison was performed to compare among treatments (letters; a: p<0.0001; b: p<0.0005; c: p<0.005; d: p<0.05).
